# Aqueous humor cytokine levels through microarray analysis and a sub-analysis based on optical coherence tomography in wet age-related macular degeneration patients

**DOI:** 10.1186/s12886-021-02152-6

**Published:** 2021-11-18

**Authors:** Jin-Ho Joo, Hyejee Kim, Jae-Ho Shin, Sang Woong Moon

**Affiliations:** 1 Department of Ophthalmology, Kyung Hee University Hospital at Gangdong, 892 Dongnam-ro, Gangdong-gu, Seoul, Republic of Korea; 2 Barunbit EYE Clinic, Seoul, Republic of Korea

**Keywords:** AMD, Aqueous humor, Cytokine, OCT

## Abstract

**Background:**

To identify disease-specific cytokine and growth factor profile differences in the aqueous humor between wet age-related macular degeneration (AMD) patients and age-matched controls and to correlate their levels with the optical coherence tomography (OCT) findings.

**Methods:**

Aqueous humors were obtained from 13 wet AMD eyes and 10 control eyes. Twenty cytokines and growth factors were measured using a RayBio antibody microarray technology in wet AMD and control eyes.

**Results:**

The samples obtained from wet AMD patients exhibited a significantly increased expression of MCP-1, MIP-1α, MIP-1β, and vascular endothelial growth factor (VEGF). Subretinal fluid (SRF) patients showed significantly lower levels of proinflammatory cytokines, such as IL-1α and GM-CSF, than those without SRF. Pigment epithelial detachments (PED) patients showed lower levels of inflammatory cytokines, such as GM-CSF, IFN-γ, and TNF-α, than those without PED. Subretinal tissue (SRT) patients showed a higher level of IFN-γ than those without SRT. Compared with the controls, type 1 macular neovascularization (MNV) patients showed increased levels of MCP-1, MIP-1α, and MIP-1β, but not VEGF (*p* = 0.083). However, type 2 MNV patients showed increased levels of MCP-1 and VEGF (*p* = 0.040 and *p* = 0.040).

**Conclusion:**

Inflammatory cytokines varied according to the type of AMD- and OCT-based parameters. Our observation of low levels of VEGF in patients with type 1 MNV implies that the inhibition of VEGF alone appears to be insufficient treatment for these patients and that cytokines such as MCP-1, MIP-1α, and MIP-1β should be modulated. And the presence of SRF in MNV may be associated with a positive prognosis because we found relatively low levels of proinflammatory cytokines.

**Supplementary Information:**

The online version contains supplementary material available at 10.1186/s12886-021-02152-6.

## Background

Wet age-related macular degeneration (AMD) is the leading cause of irreversible visual impairment in the elderly in developed countries. The involvement of vascular endothelial growth factor (VEGF) in AMD has been strongly supported in several studie s[1–3]. VEGF seems to be the major stimulus of neovascular growth originating from the retinal and choroidal vasculatur e[4]. The advent of intravitreal anti-VEGF therapy has introduced a new standard of treatment for patients with wet AM D[1]..

However, anti-VEGF treatment limitations partly arise from the profound heterogeneity found in the profiles of individual patients with choroid neovascularization (CNV) and partly arise from the insufficient effects of the anti-VEGF treatment. Although some patients may perform well with few intravitreal anti-VEGF injections, in some patients, the CNV lesion continues to progress and recur despite the monthly injection s[1, 5, 6]. Further, it is widely accepted that anti-VEGF treatment improves the vision of patients, but it may not cure or stop the disease proces s[7]. Other studies have revealed elevated concentrations of other cytokines, including VEGF, in the aqueous humor, vitreous, and retinas of eyes with neovascular disorder s[8–10]. In addition to anti-VEGF injections, the intravitreal administration of anti-inflammatory substances, such as triamcinolone, a widely used anti-inflammatory drug, has also shown positive effects in treating CNV patient s[11]. Knowledge of factors that mediate intraocular neovascular processes is important for the development of new treatment strategies.

We aimed to investigate the possible roles of various cytokines and growth factors in the pathogenesis of AMD by comparing the aqueous humor levels of 20 cytokines in eyes with cataracts and eyes with AMD. We also investigated the correlation of morphologic information based on optical coherence tomography (OCT) between cytokine information in eyes with AMD.

## Methods

This prospective trial was performed at the Department of Ophthalmology at Kyunghee University. The protocol was approved by the Ethics Committee of Kyunghee University Hospital at Gangdong (2015–07–042-014) and followed the tenets of the Helsinki protocol. Informed consent was obtained from all patients before being included in the study.

### Patient selection

We included patients with wet AMD of two subtypes of macular neovascularization (MNV); occult type, type 1 and classic type, type 2 type. Two subtypes were classified through fluorescein angiography (FA) pattern. The fluorescein angiographic appearance of classic type consists of a discrete, well-demarcated focal area of hyperfluorescence that can be discerned in the early phases of the angiogram. Occult type refers to fibrovascular pigment epithelial detachment and late choroidal-based leakage patterns on F A[12]. Only patients with recent-onset disease (treatment-naïve patients) were included in the study. All patients with wet AMD underwent comprehensive ophthalmic examinations, including best-corrected visual acuity (Snellen was converted to logMAR, Logarithm of the Minimum Angle of Resolution), slit-lamp biomicroscopy with a + 90-diopter lens, color fundus photography, FA, indocyanine green angiography, and OCT. All eyes with wet AMD had active lesions with exudative OCT changes. We excluded patients with any previous history of MNV lesion treatment, those who had undergone previous intraocular surgery (except for cataract surgery), any other type of retinal disease, glaucoma, or having an axial length > 26.5 mm.

### Control group

Controls were age-matched patients undergoing cataract surgery. We excluded patients with any type of retinal disease, glaucoma, uveitis, or a previous history of intraocular surgery.

### Acquisition of the aqueous humor samples

Aqueous humor samples were obtained at the beginning of cataract surgery in the controls and immediately before the intravitreal injection in the eyes with wet AMD via limbal paracentesis. After topical anesthesia, approximately 100 μL of aqueous humor were withdrawn using a 1-cc syringe and a 30-G needle at the limbus. The humor samples were immediately frozen and stored at − 80 °C until the cytokine measurements.

### Measurement of cytokines

We used antibody microarray technology. Samples were analyzed using RayBio® Quantikine Array kits (RayBiotech, Inc., GA, USA). These kits are used for the detection of IL-1a, IL-1b, IL-2, IL-4, IL-5, IL-6, IL-8, IL-10, IL-12p70, IL-13, granulocyte monocyte colony-stimulating factor (GM-CSF), GRO, IFN- γ, MCP-1, MIP-1α, MIP-1β, MMP-9, RANTES, TNF-α, and VEGF. A volume of 50–100 μL of undiluted aqueous humor samples was added to the arrays and these were incubated at room temperature for 1–2 h. We used these kits to identify more inflammatory cytokines, other than VEGF, in wet AMD patients. The arrays were continually washed at room temperature using gentle rocking for 20 min. The detection antibody cocktail was added to each well and incubated at room temperature for 1–2 h. We added 80 μL of Cy3 equivalent dye-conjugated streptavidin to each well, which was then incubated at room temperature for 1 h. We used an array-specific Q-Analyzer, an Excel-based program, to perform sophisticated data analysis.

### Quantitative analysis of OCT images

We measured the biomarkers in OCT images: the volume of subretinal fluid (SRF), the pigment epithelial detachments (PED), and the subretinal tissue (SRT) using Image J medical imaging software (National Institutes of Health, Bethesda, MD, USA). The boundary line for measuring the cross-sectional area of SRF, PED, and SRT in the OCT image was directly drawn by two ophthalmologists using Image J software, and the average value was measured. The boundary of the SRF was drawn along the boundary of the fluid between the sensory retina and the retinal pigment epithelium (RPE). The SRT was constructed along the boundary of the atypical fibrovascular proliferation in the subretinal space of the highly reflective region on the PRE. SRT was measured from the inner border of subretinal highly reflective material (comprising CNV, fibrosis, or hemorrhage) or fibrovascular pigment epithelial detachment or from the inner RPE layer border when no subretinal material was present, to Bruch’s membrane. (see Additional file 1 )[13] The PED was set as the boundary between the inner surface of the elevated retinal pigment epithelium and the virtual normal RPE line. After that, the volume of each morphological element was calculated by accumulating the OCT images at 240-μm intervals.

### Statistical analysis

We used Mann-Whitney U test to compare the differences between groups. Correlations between cytokines and VEGF levels were assessed using Spearman’s rank correlation. *P* values lower than 0.05 were considered statistically significant. Statistical analysis was performed using SPSS statistical package 25.0 (IBM, Armonk, New York, USA).

## Results

We included a total of 13 eyes of 13 treatment-naïve wet AMD patients that were compared to 10 age-matched control eyes of 10 participants. The demographic and baseline characteristics of the patients are listed in Table 1. The mean age of wet AMD patients and the controls was 74.2 ± 6.83 and 72.7 ± 5.12 years, respectively. There were no statistical differences regarding the age and sex distributions between the two groups (*p* = 0.364, *p* = 0.420). The mean visual acuity was 0.7 logMAR in the wet AMD group and 0.4 logMAR in the control group, showing a statistically significant difference (*p* <  0.01). From the 13 naïve wet AMD patients, 8 were type 1 (occult) MNV and 5 were type 2 (classic) MNV patients. In the wet AMD group, the number of eyes with SRF, PED, and SRT was 9, 8, and 8, respectively (Table 1).

**Table 1:** Baseline characteristics and demographics of patients with wet age-related macular degeneration (AMD) and controls

	AMD group (*n* = 13)	Control group (*n* = 10)	P*
**Mean age, years**	74.2 ± 6.83	72.7 ± 5.12	0.364
**Gender, M/F (%)**	5/8 (62.5%)	3/7 (42.9%)	0.420
**Mean baseline BCVA, logMAR (range)**	0.7 (0.18 to 2.3)	0.4 (0.00 to 1.00)	< 0.01
**AMD type (no. of eyes)**			
**Type 1**	8		
**Type 2**	5		
**Presence of SRF**	9		
**Presence of PED**	8		
**Presence of SRT**	8		

*BCVA* best corrected visual acuity, *SRF* subretinal fluid, *PED* pigment epithelial detachment, *SRT* subretinal tissue

^*^Mann–Whitney U test

### Concentration of cytokines and growth factors

**Fig. 1:**
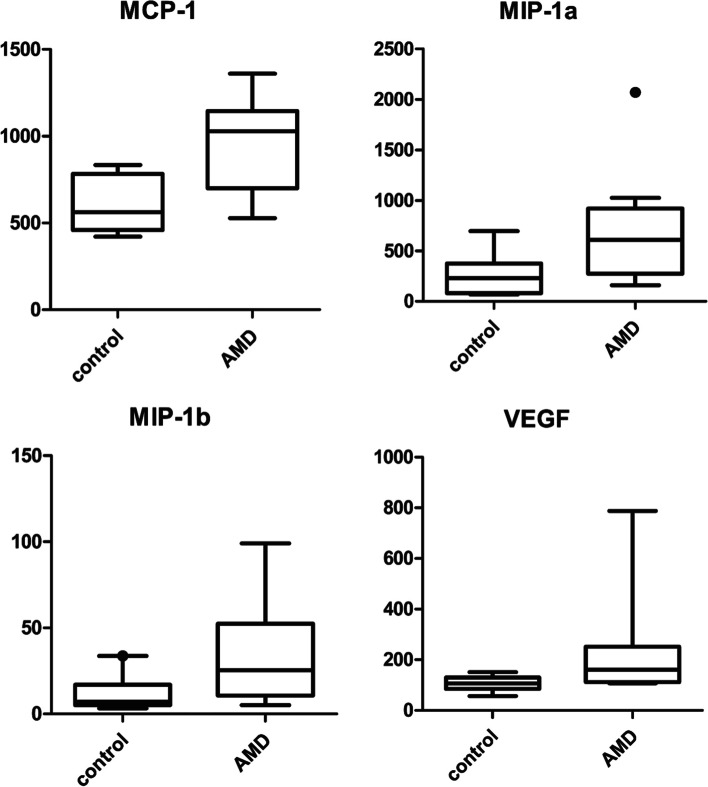
Median concentration of MCP-1, MIP-1α, MIP-1β, and vascular endothelial growth factor (VEGF) (pg/mL) in controls and patients with wet age-related macular degeneration (AMD)

Among the 20 cytokines, the levels of MCP-1 (also known as C-C motif chemokine 2; CCL2), MIP-1α (CCL3), MIP-1β (CCL4), and VEGF were significantly increased in the wet AMD group compared with the control group (*p* = 0.003, *p* = 0.041, *p* = 0.034, and *p* = 0.009) (Fig. 1). The other cytokines were also detectable in all samples but were not significantly different between patients and controls. The concentration of VEGF was correlated with the concentration of many other inflammatory cytokines, such as ILs, RANTES, and TNF-α, but not with the concentration of MCP-1 and MIP-1 s (Table 2).

**Table 2:** Cytokine profile (pg/mL, mean ± SD) and correlation between the concentration of cytokines and vascular endothelial growth factor (VEGF)

	wAMD group (*n* = 13)	Control group (*n* = 10)	p*	Correlation coefficient r	*p*†
**IL-1α**	40.54 ± 56.07	16.2 ± 11.88	0.278	0.46	**0.028**
**IL-1β**	7.6 ± 16.30	2.3 ± 3.69	0.734	0.76	**0.001**
**IL-2**	17.5 ± 12.99	11.6 ± 7.82	0.395	0.44	**0.038**
**IL-4**	10.3 ± 13.6	6.6 ± 4.04	0.962	0.56	**0.009**
**IL-5**	8.8 ± 7.99	9.2 ± 4.92	0.285	0.40	0.058
**IL-6**	24.4 ± 10.65	33.1 ± 11.19	0.095	0.64	**0.002**
**IL-8**	29.6 ± 32.62	19.1 ± 8.87	0.427	0.36	0.088
**IL-10**	2.6 ± 1.83	2.6 ± 1.06	0.792	0.70	**0.001**
**IL-12p70**	3.7 ± 4.77	2.3 ± 1.35	0.734	0.43	**0.047**
**IL-13**	9.3 ± 4.80	10.2 ± 2.72	0.193	0.52	**0.014**
**GM-CSF**	28.3 ± 12.98	33.6 ± 20.71	0.734	0.37	0.076
**GRO**	457.4 ± 215.28	381.1 ± 149.52	0.277	0.23	0.272
**IFN-γ**	293.1 ± 103.77	317.3 ± 114.59	0.651	0.21	0.329
**MCP-1**	**951.8 ± 265.13**	**540.3 ± 261.01**	**0.003**	−0.03	0.903
**MIP-1α**	**679.1 ± 525.50**	**263.3 ± 226.85**	**0.041**	−0.51	0.807
**MIP-1β**	**32.9 ± 27.1**	**11.4 ± 10.1**	**0.034**	0.29	0.180
**MMP-9**	151.4 ± 111.58	108.1 ± 79.15	0.343	0.18	0.393
**RANTES**	2.9 ± 3.76	0.9 ± 0.59	0.343	0.65	**0.002**
**TNFα**	977.0 ± 401.35	1140.8 ± 377.42	0.310	0.44	**0.038**
**VEGF**	**236.2 ± 196.31**	**106.3 ± 30.41**	**0.009**	–	–

*wAMD* wet age-related macular degeneration

^*^Mann-Whitney U test

^†^Spearman’s rank correlation

### Intraocular cytokines and OCT-based retinal morphology in wet AMD patients

**Fig. 2:**
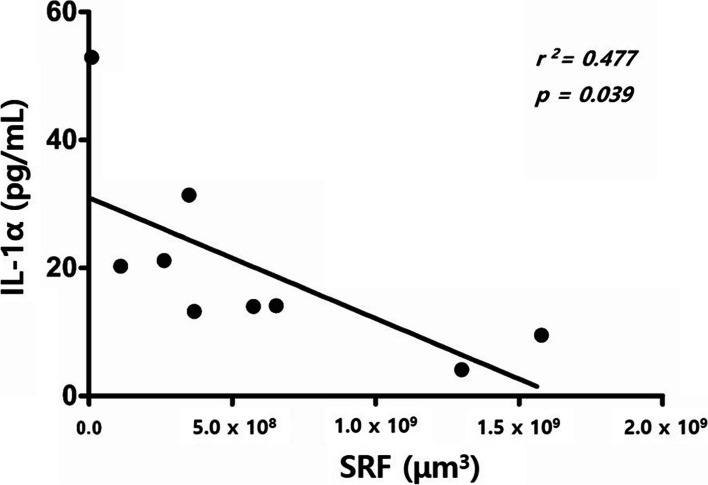
Correlation between the volume of subretinal fluid (SRF) and IL-1α. There is a negative correlation between the level of IL-1α and the volume of SRF in the SRF-positive group

The wet AMD patient groups were subdivided according to the OCT components. There were nine wet AMD patients with SRF and four wet AMD patients without SRF. The nine patients with MNV and SRF showed significantly lower levels of proinflammatory cytokines, such as IL-1α and GM-CSF, compared with patients with MNV without SRF (*p* = 0.036, and *p* = 0.05). Moreover, it was confirmed that there was a statistically significant negative correlation between the volume of SRF and the concentration of IL-1α (Fig. 2).

Similar phenomena were also observed in patients with PED. Patients with PED showed relatively low levels of inflammatory cytokines, such as GM-CSF, IFN-γ, and TNF-α, compared with those without PED (*p* = 0.006, *p* = 0.034, and *p* = 0.011). In contrast, patients with SRT showed a relatively high level of IFN-γ compared with those without SRT (*p* = 0.045) (Table 3). There was no statistically significant correlation between the volume of PED and SRT and the cytokine levels.

**Table 3:** Levels of cytokine (pg/mL, mean ± SD) in the aqueous humor in wet age-related macular degeneration patients

	SRF (*n* = 9)			PED (*n* = 8)			SRT (*n* = 8)		
	(+)	(−)	P*	(+)	(−)	P*	(+)	(−)	P*
IL-1α	18.5 ± 15.9	216.3 ± 199.5	**0.036**	15.1 ± 9.7	170 ± 192.6	0.622	90.8 ± 152.1	17.9 ± 8.7	0.833
GM-CSF	19.9 ± 11.4	71.0 ± 67.4	**0.050**	18.5 ± 10.9	72.9 ± 65.6	**0.006**	48.1 ± 50.6	14.7 ± 13.4	0.065
IFN-γ	264.6 ± 62.6	676.6 ± 611.8	0.076	262.5 ± 63.2	681.4 ± 607.3	**0.034**	487.0 ± 451.5	238.4 ± 43.2	**0.045**
MCP-1	984.1 ± 255.7	854.4 ± 266.1	0.503	964.3 ± 228.3	899.1 ± 343.3	0.284	963.8 ± 287.6	913.0 ± 710.1	0.622
MIP-1α	812.8 ± 545.3	330.9 ± 127.6	0.076	787.4 ± 561.3	388.1 ± 190.6	0.065	506.3 ± 276.0	917.7 ± 710.1	0.284
MIP-1β	38.2 ± 29.1	21.3 ± 13.0	0.414	34.0 ± 29.2	30.7 ± 20.2	0.724	27.5 ± 17.4	41.9 ± 36.5	0.622
TNFα	860.0 ± 225.2	1973.3 ± 1399.3	0.106	836.4 ± 216.7	2026.6 ± 1343.3	**0.011**	1409.4 ± 1100.2	871.6 ± 295.3	0.284
VEGF	189.2 ± 73.6	349.3 ± 327.7	0.076	165.0 ± 57.6	403.8 ± 289.9	0.354	283.7 ± 232.8	166.0 ± 60.5	0.524

*VEGF* vascular endothelial growth factor

^*^Mann-Whitney U test

### Levels of cytokines according to wet AMD classification

We classified patients with wet AMD into two groups. Eight eyes of eight patients were classified as type 1 MNV, and five eyes of five patients were classified as type 2 MNV. When the OCT parameters were compared in the type 1 and type 2 MNV groups, the average SRF volume value was larger in the type 1 MNV group, and the PED volume, SRT volume and central macular thickness (CMT) were larger in the type 2 MNV group. However, all these parameters did not show a statistically significant difference. (Table 4).

**Table 4:** Comparison of optical coherence tomography (OCT) parameters between type 1 and type 2 macular neovascularization (MVN)

	Type 1 MNV (*n* = 8)	Type 2 MNV (*n* = 5)	P*
**SRF volume (**μm^**3**^**)**	505.04 × 10^6^ ± 373.83 × 10^6^	315.56 × 10^6^ ± 472.61 × 10^6^	0.284
**PED volume (μm**^**3**^**)**	1151.58 × 10^6^ ± 2517.02 × 10^6^	1711.82 × 10^6^ ± 3111.73 × 10^6^	0.524
**SRT volume (μm**^**3**^**)**	1655.24 × 10^6^ ± 2258.04 × 10^6^	2238.47 × 10^6^ ± 3028.61 × 10^6^	0.448
**CMT (μm)**	377.00 ± 91.49	431.00 ± 150.29	0.724

*SRF* subretinal fluid, *PED* pigment epithelial detachment, *SRT* subretinal tissue, *CMT* central macular thickness

^*^Mann–Whitney U test

Compared with the control group, the type 1 MNV group showed increased levels of MCP-1, MIP-1α, and MIP-1β, (*p* = 0.001, *p* = 0.02, and *p* = 0.012), but not of VEGF (*p* = 0.083). Meanwhile, the type 2 MNV group showed increased levels of MCP-1 and VEGF (*p* = 0.040, and *p* = 0.040). Patients with type 1 MNV showed relatively low concentrations of INF-γ and TNF-α compared to those with type 2 MNV (*p* = 0.006, and *p* = 0.019) (Table 5).

**Table 5:** Levels of significant cytokines (pg/mL, mean ± SD) in the aqueous humor among the 3 groups

	Control (*n* = 10)	Type 1 (*n* = 8)	Type 2 (*n* = 5)
**IFN-γ**	317.3 ± 114.59	247.3 ± 46.7	621.9 ± 542.5
*p value* versus *controls*	–	0.460	0.129
*p value* versus *type 1*	–	–	**0.006**
**MCP-1**	540.3 ± 261.01	1003.5 ± 209.2	849.4 ± 317.3
*p value* versus *controls*	–	**0.001**	**0.040**
*p value* versus *type 1*	–	–	0.284
**MIP-1α**	263.3 ± 226.85	842.1 ± 573.8	380.4 ± 166.0
*p value* versus *controls*	–	**0.021**	0.206
*p value* versus *type 1*	–	–	0.065
**MIP-1β**	11.4 ± 10.1	37.2 ± 29.5	26.3 ± 20.1
*p value* versus *controls*	–	**0.012**	0.129
*p value* versus *type 1*	–	–	0.724
**TNFα**	1140.8 ± 377.42	836.4 ± 231.6	1788.4 ± 1279.4
*p value* versus *controls*	–	0.274	0.440
*p value* versus *type 1*	–	–	**0.019**
**VEGF**	106.3 ± 30.41	168.8 ± 60.3	350.0 ± 278.4
*p value* versus *controls*	–	0.083	**0.040**
*p value* versus *type 1*	–	–	0.354

*VEGF* vascular endothelial growth factor

^*^Mann-Whitney U test

## Discussion

We investigated various cytokine and angiogenic factors associated with aqueous humor. The vitreous sample would be more appropriate than the aqueous sample because it better reflects the state of the retina in AMD patients. Nevertheless, obtaining samples from the vitreous is dangerous and may cause adverse effects, such as vitreous hemorrhage, retinal detachment, endophthalmitis, among others. There may be differences in the concentration of immune mediators between the aqueous and the vitreous humor. However, the collection of samples from the aqueous humor is safer and easier, and similar levels have been reported in both the aqueous and vitreous humor s[14–16]. Immune mediators in the aqueous humor likely explain the presence of immune mediators in the vitreous humor. Thus, the results of our study suggest that the eyes of patients with wet AMD showed increased MCP-1, MIP-1α, MIP-1β, and VEGF concentrations in the vitreous.

MCP-1 is a potent chemotactic factor for monocytes and macrophages, and plays a critical role in angiogenesis and inflammatory processe s[17]. MCP-1 attracts macrophages into the CNV lesion and assists with digestion of the RPE and Bruch’s membrane in the experimental model s[18, 19]. MCP-1 also induces angiogenesis by recruiting other cells, such as tumor-associated macrophages. These cells, in turn, release growth and angiogenic factors, such as VEG F[20]. Our results showed significantly elevated aqueous humor levels of MCP-1 in patients with CNV versus controls and were in agreement with those of previous studies. MIP-1α and MIP-1β have been implicated in retinal inflammation, particularly in early T-cell-dependent stage s[21–23]. Their production by T lymphocytes entering the tissue is advantageous in many inflammatory situations because they amplify inflammatory cell recruitment. However, this cascade effect is likely to exacerbate predisposing factors in susceptible individual s[24]. Consequently, T cell regulation of MIP-1α and MIP-1β production is important. An in vitro study has shown that RPE cells downregulate the levels of CCL3(MIP-1α) and CCL4(MIP-1β) production by T lymphocytes using the soluble mediators sCD54 and prostaglandin E2 (PGE2 )[24]. Yang et al. found that MIP-1a expression increased in the corneal neovascularization tissue of mice, suggesting that MCP-1 and MIP-1a is related to inflammation and neovascularizatio n[25]. In addition, it was confirmed that MIP-1a was significantly increased in a study targeting the laser-induced CNV mouse model. MIP-1a was significantly correlated with the CNV lesion area, suggesting that the migration of MIP-1a-mediated macrophages could induce an inflammatory response and affect CNV formatio n[26]. Our novel results showed elevated aqueous humor concentrations of MIP-1α and MIP-1β in eyes with wet AMD. We believe that these findings are attributed to a disrupted RPE cell regulation because of degeneration and suggest that inflammatory factors may influence the pathogenesis of AM D[27]..

Quantitative analysis of OCT images is useful for the treatment strategy selection and for monitoring the biological response to treatment. It is possible to characterize individual MNV lesions according to the presence or number of sub-components. This could help investigate MNV heterogeneous lesions. Unfortunately, there is a lack of a reliable correlation between the role of intraocular cytokines and OCT-based parameters. We analyzed the differences in cytokine levels between wet AMD patients with SRF or PED and those without SRF or PED.

As described in the results, patients with MNV and SRF showed low concentrations of proinflammatory cytokines, such as IL-1α and GM-CSF. IL-1α is a proinflammatory cytokine that derives macrophages and induces acute inflammatio n[28]. Pathologically, IL-1α can stimulate RPE cells to secrete GM-CSF, which is a potent chemoattractant that recruits macrophages to the retina. IL-1α is also implicated in the pathogenesis of CNV. Blocking IL-1α receptors could inhibit the development of CNV in an experimental animal mode l[29]. Therefore, lower intraocular concentrations of IL-1α indicate lower levels of inflammation in CNV pathogenesis. Our results indicate that SRF may be associated with a positive prognosis because we found relatively low levels of proinflammatory cytokines in the SRF-positive group. Other studies have found that SRF may be associated with a benign disease course in patients with CNV lesion s[7]. Moreover, in the SRF-positive group, we found a negative correlation between the concentration of IL-1α and the volume of SRF (Fig. 2). A potential explanation for this correlation is that SRF could be suggestive of a functional providing RPE and photoreceptor survival, in contrast to vascular atrophy in the sub-RPE space. The presence of SRF may be suggestive of a less aggressive, perhaps even supportive, stage of CNV, rather than advanced destructive neurosensory ingrowth associated with intraretinal exudation. This is supported by the fact that treatment-refractory SRF was not detrimental to the vision outcome in a recent stud y[7]. Interestingly, positive effects of SRF on the visual outcome have also been reported in diabetic macular edema and retinal vein occlusio n[30, 31]..

In the presence of PED on OCT, it was confirmed that IFN-γ and TNF-α values were significantly lower than those of the group without PED. In other animal studies, it has been argued that they may help maintain the outer blood-retinal barrier by providing fluid transport in RPE cell s[32, 33]. Therefore, through the results of this study, it can be considered that PED may occur due to a decrease in these cytokines. Significantly smaller values were also measured in the type 1 MNV group than in the type 2 MNV group, and it is thought that additional research is needed on this result.

Our findings suggest that angiographic classification may be an important clue to determine the treatment strategy. Patients with type 1 MNV showed increased levels of MCP-1, MIP-1α, and MIP-1β, but not VEGF, compared to those with type 2 MNV. These results suggest that in patients with type 1 MNV, inhibition of VEGF alone may not be a sufficient treatment. The levels of VEGF and angiogenic inflammatory cytokines were not significantly increased in patients with type 1 MNV compared with the control group. Inhibition of the MCP-1, MIP-1α, and MIP-1β related cascade may be a promising advance in MNV treatment, especially for patients with type 1 MNV. Considering that platelet-derived growth factor (PDGF) is the main growth factor that stimulates the secretion of MCP-1,[34] PDGF inhibition could be a promising treatment for MNV, particularly in patients with type 1 MNV.

Moreover, we confirmed that the VEGF concentration of aqueous humor in type 2 MNV was higher than that of type 1 MNV or in the control group. It is thought that type 2 MNV patients have a higher VEGF concentration in the vitreous cavity and anterior chamber due to the growth of neovascularization in the subretina, and that their prognosis is worse. In fact, several studies also found that, after anti-VEGF injection treatment, type 2 MNV patients showed worse visual acuity and a poor responsiveness to injection when compared to type 1 MNV patient s[35, 36]..

A limitation of this study is that the sample size was small; therefore, a larger multicenter prospective randomized study is required to clarify the effects of inflammatory factors in AMD. In addition, it would be good to analyze the sample size larger to confirm the correlation between quantitative data of OCT and aqueous humor cytokine. Moreover, since factors such as SRF and PED may be temporary, additional studies are needed to confirm the change in cytokine concentration according to changes in these factors after injection treatment in the same patient.

## Conclusions

In conclusion, inflammatory cytokines varied according to the type of AMD- and OCT-based parameters. Our observation of low levels of VEGF in patients with type 1 MNV implies that the inhibition of VEGF alone appears to be insufficient treatment for these patients and that cytokines such as MCP-1, MIP-1α, and MIP-1β should be modulated. And the presence of SRF in MNV may be associated with a positive prognosis because we found relatively low levels of proinflammatory cytokines. Understanding the pathological mechanisms of angiogenesis and the cytokines involved is necessary to identify new therapeutic targets for patients with neovascular diseases.

## Supplementary Information

**Additional file 1.** Spectral-domain optical coherence tomography scan showing subretinal tissue. Spectral-domain optical coherence tomography scan showing subretinal tissue (SRT), which includes subretinal highly reflective material and fibrovascular RPE detachment.

## Data Availability

Not applicable.

## Abbreviations

AMD: Age-related macular degeneration
VEGF: Vascular endothelial growth factor
CNV: Choroidal neovascularization
OCT: Optical coherence tomography
MNV: Macular neovascularization
FA: Fluorescein angiography
logMAR: Logarithm of the Minimum Angle of Resolution
SRF: Subretinal fluid
PED: Pigment epithelial detachments
SRT: Subretinal tissue
RPE: Retinal pigment epithelium
CMT: Central macular thickness
PDGF: Platelet-derived growth factor
