# Supplementary material for: Aqueous humor cytokine levels through microarray analysis and a sub-analysis based on optical coherence tomography in wet age-related macular degeneration patients
Source: BMC Ophthalmol. 2021 Nov 18;21:399. doi: 10.1186/s12886-021-02152-6 (PMC8603589; doi:10.1186/s12886-021-02152-6)

**<Additional materials>**

**Aqueous humor cytokine levels through microarray analysis and a sub-analysis based on optical coherence tomography in wet age-related macular degeneration patients**

**Jin-Ho Joo^1^, Hyejee Kim^2^, Jae-Ho Shin^1^, Sang Woong Moon^1^**

^1^Department of Ophthalmology, Kyung Hee University Hospital at Gangdong, Seoul, Republic of Korea

^2^Barunbit EYE clinic, Seoul, Republic of Korea

**Additional file 1.** Spectral-domain optical coherence tomography scan showing subretinal tissue (SRT), which includes subretinal highly reflective material and fibrovascular RPE detachment.


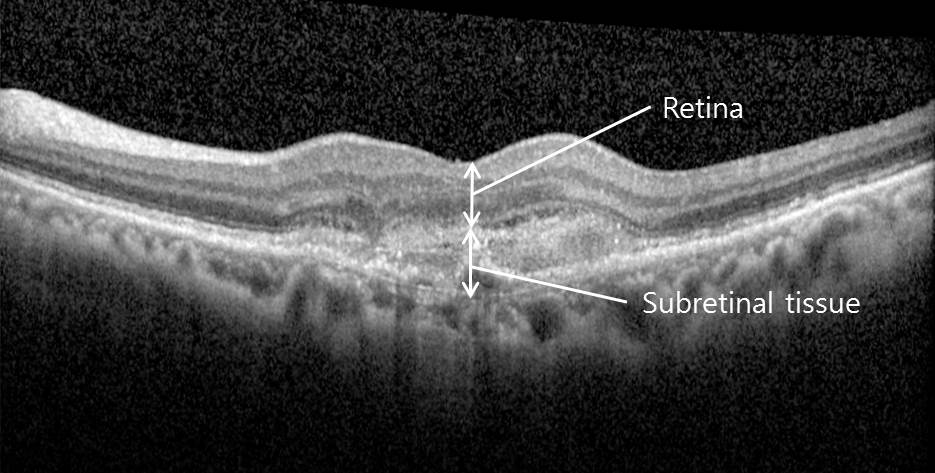

Supplement: Supplementary file 1 — Additional file 1. Spectral-domain optical coherence tomography scan showing subretinal tissue. Spectral-domain optical coherence tomography scan showing subretinal tissue (SRT), which includes subretinal highly reflective material and fibrovascular RPE detachment. [file 12886_2021_2152_MOESM1_ESM.docx]
